# The Role of Neutrophil Gelatinase-Associated Lipocalin (NGAL) as a Biomarker in Predicting the Function of Kidney Grafts: A Literature Review

**DOI:** 10.3390/jcm15176803

**Published:** 2026-09-02

**Authors:** Alexander A. Dermanis, Dominic Thompson, Georgios Koimtzis

**Affiliations:** 1 Department of General Surgery, Glangwili Hospital, Hywel Dda Health Board, Carmarthen SA31 2AF, UK; 2 Department of Transplant Surgery, University Hospital of Wales, Cardiff CF14 4XW, UK; drgxkoimtzis@gmail.com

**Keywords:** biomarker, transplantation, outcomes

## Abstract

**Background/Objectives**: Kidney transplantation is the gold standard treatment for end-stage renal failure. Numerous studies have sought to identify biomarkers that can predict early outcomes. Neutrophil gelatinase-associated lipocalin (NGAL) is a biomarker that is hypothesised to be an important predictor of early surgical outcomes. **Methods**: A targeted narrative search was performed using prespecified search terms. Reference lists were searched to identify and yield more articles. **Discussion**: This review has identified several domains in which urinary and serum NGAL may be useful as well as limitations in its application. **Conclusions**: NGAL is a promising biomarker; however, further research is necessary to establish best practice in its use in the early post-operative phase.

## 1. Introduction

Kidney transplantation remains the gold standard treatment for patients with end-stage renal failure [1]. Early post-operative outcomes remain excellent, and the number of transplants performed annually is increasing [2]. Studies focusing on long-term outcomes have demonstrated that poor early post-operative outcomes can be a predictor of a worse long-term outcome [3,4]. Early graft outcomes therefore form a quality metric that can be used in the evaluation of the standard of care provided by a transplant service [5]. These outcomes include but are not limited to 30-day morbidity, 30-day mortality, acute rejection and delayed graft function [5]. Consequently, the need to address quality metrics remains an important issue in determining short-term and long-term patient outcomes.

Delayed graft function (DGF) is a condition characterised by the failure of the kidney graft to function or produce urine and therefore is typically defined as the need for dialysis within seven days following transplantation [6]. However, this definition is not universally applied, and more than ten variant definitions exist in the literature [7], including: creatinine-based definitions, composite definitions combining dialysis requirement with creatinine trajectory and severity-staged definitions that categorise DGF by duration (e.g., mild, moderate, severe) rather than as a binary outcome. It is hypothesised that DGF occurs with interruption of blood flow to the graft following procurement and cooling, with subsequent reperfusion leading to oxidative stress and acute tubular necrosis [8]. Multiple studies have indicated it can be associated with poorer short-term and long-term outcomes, including long-term survival, and it therefore remains an important surgical quality metric [3,5,9,10].

Early recognition of DGF, prevention and management in the form of supportive care to the graft are paramount to prevent graft failure or failure to rescue [11]. A number of risk factors have been identified for DGF, but their individual sensitivity in predicting DGF is limited [12,13]. This is partly due to a complex interplay of multiple factors including donor, patient and graft characteristics in what leads up to this complication [14]. Whilst risk scores have attempted to address this issue, constraints in validation of these models are in part driven by differences in criteria and decision making between different centres on dialysis, which ultimately affects classification and thus the reported incidence of DGF, therefore limiting these scoring systems’ ability to uniformly predict delayed graft function across populations [13,15]. Therefore, research is underway to assess whether there are specific biological markers that can predict a delay in graft function and improve its prediction. Neutrophil gelatinase-associated lipocalin (NGAL) is a biomarker that has gained traction in recent years for its potential diagnostic use [16]. This review aims to characterise NGAL, evaluate the available literature on this biomarker, and suggest future research and study designs to evaluate its utility in predicting DGF and other transplantation outcomes.

## 2. Methods

A narrative review of the available studies was planned with a targeted literature search in the PubMed, OVID Medline, Google Scholar and Web of Science databases from January 2000 to January 2026. Search terms used in this search included the following terms: “NGAL” OR “neutrophil gelatinase-associated lipocalin” OR “lipocalin-2” OR “LCN2” OR “siderocalin” OR “uterocalin” OR “oncogene 24p3” OR “24p3”AND “kidney transplant*” OR “renal transplant*” OR “kidney graft*” OR “renal graft*” OR “kidney allograft*” OR “renal allograft*” OR “acute kidney injury”. Studies were included if they assessed outcomes of NGAL in patients receiving a kidney graft. Studies were prioritised for inclusion based on their level of evidence, with randomised controlled trials given priority for inclusion. Studies were excluded if they (i) did not report outcomes for NGAL, (ii) were based on non-human subjects, or (iii) were conference abstracts, editorials, or letters to the editor. Studies addressing NGAL in non-transplant patients were generally excluded; however, selected studies were included to allow contextualization in its discovery and assessment across different cohorts. The literature search and selection of studies were performed by two co-authors (AD and DT), with disagreements resolved by consulting the senior author (GK). In total, 5123 articles were identified, and after assessment, 25 studies were included in the final manuscript.

## 3. Discovery of Neutrophil Gelatinase-Associated Lipocalin (NGAL)

Neutrophil gelatinase-associated lipocalin (NGAL), which is also known as siderocalin, uterocalin or oncogene 24p3, was first described by a Danish research team in 1993 [17]. It is a protein composed of 178 amino acids with a molecular weight of 25 kDa [16]. It is expressed by cardiomyocytes, kidney, lung and ileal epithelial cells [18]. It plays a role as an acute phase protein but is also known to interact in the process of oncogenesis [19], apoptosis [20] and may have an immunological role in inhibiting bacterial growth [21]. Interest in NGAL piqued when it was shown to be secreted from the kidney tubules in response to ischaemic injury and therefore was a potential biomarker that could indicate an acute kidney injury (AKI) [22]. It was therefore hypothesised that NGAL could potentially act as an early tracer for kidney injury before meaningful rises in serum creatinine [23].

## 4. NGAL in Kidney Disease

Multiple early studies focused on the role of NGAL in AKI. A prospective cohort study in 2007 by Zappitelli et al. assessed the use of urinary NGAL to diagnose AKI in critically unwell children [24]. They found that in 140 children studied, urinary NGAL concentrations increased at least sixfold higher than in controls, 2 days before a 50% or greater rise in serum creatinine, indicating that urinary NGAL testing could lead to earlier prediction of acute kidney injury. A 2014 study reported that of the 77 patients assessed, 45 developed AKI; most of these patients had elevated urinary NGAL, and plasma NGAL in the first 6 to 24 h post-presentation [25]. Nevertheless, the evidence was not limited to acute kidney injury; other studies also found that NGAL can predict progression of chronic kidney disease (CKD); a 2009 study found that both serum and urinary NGAL levels were inversely correlated with eGFR [26]. Following this, evidence to support NGAL in the diagnosis of AKI in patients with acutely decompensated states such as heart failure emerged. A 2015 randomised pilot study by Lai et al. identified that elevated NGAL levels in patients presenting with acute heart failure were significantly correlated with AKI (area under the curve [AUC]: 0.81, 95%CI: 0.76–0.88, *p* < 0.001) [27]. Interestingly, NGAL levels greater than 170 ng/mL also indicated an increased risk of cardiac mortality (HR 1.77, 95%CI: 1.24–2.83, *p* = 0.01).

## 5. Utility of NGAL in Diagnosis of Delayed Graft Function

Given the evidence supporting NGAL in predicting AKI, progression of CKD and long-term prognosis in decompensated states, attention turned to its utility in kidney transplantation, specifically in determining early surgical outcomes, such as delayed graft function. Early studies indicated that NGAL levels were present in the urine and plasma at 6 and 12 h after transplantation, respectively, [28] and several studies sought to establish whether serum and plasma NGAL are specific and sensitive biomarkers for predicting DGF in patients post-transplantation [29,30,31,32].

### 5.1. Plasma NGAL

In their prospective study of plasma NGAL in 41 consecutive patients, Bataille et al. found that for patients diagnosed with DGF, NGAL levels were significantly higher at 12 h post-transplantation (571 (467–634) vs. 242 (158–299) ng/mL, *p* < 0.0001) and at day 1 post-transplantation (466 (356–627) vs. 165 (91–248) ng/mL, *p* < 0.0001) when compared with controls [29]. Similarly, Cantaluppi et al. prospectively evaluated NGAL levels in 50 kidney transplant patients and found that mean NGAL levels in the DGF group were significantly different compared with the non-DGF group (662.7 ± 97.2 vs. 379.7 ± 139.7 ng/mL, *p* < 0.001) [30]. They also found that in the non-DGF group, NGAL was able to distinguish between slow graft function and immediate graft function with statistical significance (444.1 ± 149.5 ng/mL versus 307.8 ± 84.5 ng/mL, *p* < 0.001). Based on their findings, they then generated an ROC curve and they identified plasma NGAL levels of 532 ng/mL as the most sensitive and specific threshold to predict DGF (AUC 0.94; sensitivity 90.9%; specificity 80.6%). This led them to conclude that serum NGAL measurements are an accurate and early biomarker of graft function. Both studies are limited by their small sample sizes, which lowers the certainty of the conclusions presented.

### 5.2. Serum NGAL

Despite the cut-offs identified by Cantaluppi et al. for plasma NGAL, other studies have found lower thresholds for serum NGAL, such as in the case of Ramizeh et al., who identified a cut-off of 174 ng/mL for sensitivity and specificity of 100% and 95.5%, respectively [31], and Lee et al., which identified a cut-off of 233.3 ng/mL for sensitivity of 76.6% and specificity of 77.8%, respectively [32]. Given that serum NGAL rises within the first 12 h, timing of NGAL measurement is important in determining the cut-off, such as in Pezeshgi et al.’s study of 37 patients, where they determined at 12 h the cut-off value for serum NGAL to be 309 ng/mL, with a sensitivity of 100% and specificity of 92%, respectively [33]. Nevertheless, the low sample sizes temper any comparison in diagnostic accuracy estimates and should be interpreted with caution. Whilst early suspicion of DGF is important to ensure timely clinical management, the optimum timing for detection needs to be balanced with the optimal sensitivity and specificity. Therefore, whilst studies mostly indicate strong sensitivity and specificity for serum NGAL predicting DGF, consensus is needed to establish the ideal timing and cut-off value for testing serum NGAL levels in order to predict DGF.

### 5.3. Urinary NGAL

Given that urine NGAL can also be detected within 6 h following transplantation [28], it forms an alternative approach that could potentially offer an earlier diagnosis of DGF when compared with serum NGAL. In their 2013 prospective cohort study of 40 kidney transplant recipients, Fonseca et al. collected serial urine NGAL at different time points to assess their predictive value in identifying DGF [34]. At 3–6 h, 1 day, 2 days and 4 days urine NGAL levels were each independently correlated with the development of DGF (OR_3–6 h_ 1.15, *p* = 0.04/OR_day 1_ 1.22, *p* = 0.012/OR_day 2_ 1.35, *p* = 0.004/OR_day 4_ 3.01 *p* = 0.035). Similarly, Cui et al. also measured serial urine NGAL at 4 h, 12 h, 24 h and 48 h and found that there was a significant difference favouring those who went on to develop DGF (median_4 h_ 917.1 vs. 356.6, *p* = 0.018/median NGAL_12 h_ 850.6 vs. 363.3 ng/mL, *p* = 0.001/median NGAL_1 day_ 959.2 vs. 307.1 ng/mL, *p* = 0.010/median NGAL_2 day_ 665.6 vs. 37.9 ng/mL, *p* = 0.009) [35].

Whilst measurement of urinary NGAL may lead to early suspicion, the caveat is that some studies show it may have lower specificity and sensitivity at early time points, with reported sensitivity at time points before 24 h ranging from 77% to 80% and specificity ranging from 68.7% to 88% [33,34]. At the 24 h time point, sensitivity and specificity are more similar to serum NGAL. For example, in their study of 124 patients, by measuring urine NGAL at day 1, Lacquaniti et al. were able to demonstrate for a cut-off of 97 ng/mL, a sensitivity and specificity of 71.8% and 100% in predicting DGF [36]. They identified that elevated urinary NGAL levels were associated with a 10% increased risk of DGF (HR: 1.10; 95%CI: 1.04–1.25; *p* < 0.001). Similar studies have found higher cut-off values; Hall et al., for example, reported a cut-off of 350 ng/mL for a sensitivity of 77% and specificity of 74% [37]. Similarly to serum NGAL, the ideal timing and cut-off for measuring urinary NGAL still remain contentious.

## 6. Utility of NGAL in Predicting Duration of DGF

### 6.1. Urinary NGAL

Several studies have sought to correlate urine NGAL levels with the duration of DGF in order to identify if patients are at a higher risk of requiring prolonged graft support. In their 2025 study, de Rooij et al. sought to assess whether urinary NGAL can be used as an alternative to ^99m^Tc-MAG3 renography to predict the duration of DGF [38]. They compared the outcomes of 89 donation after cardiac death (DCD) transplant recipients. Predictive performance was compared with the area under the curve (AUC) from receiver operating curve analyses. They categorised DGF into mild (≥7–14 days), moderate (≥14–<21 days) and severe (≥21 days). At post-operative day 4, they identified that urinary NGAL outperformed renography and other biomarkers in predicting DGF with AUCs of 0.97 (95%CI: 0.90–1.00) and 0.92 (95%CI: 0.86–0.98), respectively. At post-operative day 10, fractional NGAL excretion, when compared with renography, better predicted severe DGF with AUCs of 0.74 (95%CI: 0.55–0.93) versus 0.65 (95%CI: 0.48–0.82) respectively. They therefore concluded that not only can NGAL predict the formation of DGF, but it can also comparably predict the severity of DGF when compared with a gold standard (in this case ^99m^Tc-MAG3 renography).

### 6.2. Serum NGAL

For serum NGAL, studies have sought to establish a correlation between NGAL levels and duration of DGF. In their 2015 study of 20 kidney transplant DCD recipients, Van den Akker et al. sought to assess whether serum NGAL can predict duration of delayed graft function [39]. By measuring serum NGAL at different post-operative time points, they found a correlation between serum NGAL levels and duration of DGF for day 1 (*p* = 0.004, r^2^ = 0.19), day 4 (*p* = 0.02, r^2^ = 0.28) and day 7 (*p* = 0.03, r^2^ = 0.27). This led the authors to conclude that NGAL as an early biomarker can predict duration of DGF. High-quality data correlating NGAL with duration of delayed graft function are lacking, particularly when considering the illness state of the patient may vary depending on the duration and severity of DGF and the low sample sizes of the studies presented here.

## 7. NGAL as a Long-Term Prognostic Factor of Graft Function

### 7.1. Urinary NGAL

Given that acute kidney injury and delayed graft function can be considered as prognostic indicators for long-term renal function [40,41,42], it is therefore hypothesised that NGAL itself could potentially form a prognostic marker for long-term graft function. Whilst long-term data are lacking, a few studies comment on the predictive value of NGAL in long-term renal function. In a multivariable regression analysis adjusting for confounders, Fonseca et al. correlated uNGAL measured on the fourth and seventh days with one-year graft function (regression coefficient_day 4_ 0.067, 95%CI: 0.002–0.132, *p* = 0.045; regression coefficient_day 7_ 0.138, 95%CI: 0.041–0.235, *p* = 0.007) [34]. Similarly, Lacquaniti et al. found that elevated urine NGAL levels on day 1 were associated with a 15% risk of allograft nephropathy progression (HR: 1.15; 95%CI: 1.09–1.26; *p* < 0.001) [36]. Whilst these studies support early biomarker measurement, measurements of NGAL in the outpatient setting can also predict graft survival. Kielar et al. assessed urinary NGAL levels in 109 patients at least one year post-transplantation to identify if NGAL can predict changes in renal function [43]. By using a multiple regression model, they found that urinary NGAL levels are an independent predictor of change in eGFR (β = −0.22 ± 0.10, *p* = 0.039) as well as long-term mean eGFR (β = −0.11 ± 0.04, *p* = 0.008). Additionally, in a logistic regression analysis they identified that urinary NGAL was also an independent predictor of urinary tract infection during follow-up (OR = 3.46, 95%CI 1.29–9.21, *p* = 0.012), after adjustment for sex.

### 7.2. Serum NGAL

In their 2024 study of 709 patients, Swolinsky et al. aimed to assess serum NGAL levels at 2 months following transplantation and correlate this with graft loss to establish whether outpatient measurement of NGAL can predict graft loss [44]. In their multivariate analysis, they found that serum NGAL was strongly correlated with death-censored graft loss (HR 3.23, 95%CI: 1.93–5.41, *p* < 0.0001); however, it did not outperform eGFR either when combined as a risk model or compared independently.

### 7.3. Plasma NGAL

In 2019, Nielsen et al. performed a post hoc analysis where they correlated serum NGAL with early and late graft outcomes [45]. Of the 199 patients who provided a plasma NGAL sample, the area under the curve was 0.91 ± 0.02 for DGF, supporting an association between plasma NGAL and DGF; however, the correlation between day 1 plasma NGAL and eGFR at 1 year for the 180 patients studied was weak and not statistically significant (r^2^ 0.01, *p* = 0.07).

## 8. NGAL as a Composite Measure

Some authors have proposed using a combination of different biomarkers to predict outcomes. In their analysis, Cui et al. considered whether various combinations of urine NGAL with interleukin-18 (Il-18) and serum creatinine (sCr) could accurately predict DGF [35]. In the ROC curve displayed, the area under the curve for NGAL + Il-18 was reported as 0.844 (0.699–0.937), for NGAL + sCr was 0.978 (0.831–0.995), and for NGAL + Il-18 + sCr was 0.984 (0.887–0.994), indicating that using a composite of biomarkers had strong diagnostic performance. Whilst data on NGAL use as a composite assay are limited, other potential biomarkers for consideration in a combination assay include lactate dehydrogenase (LDH), glutathione S-transferase, N-acetyl-beta-D-glucosaminidase, kidney injury molecule-1, liver-type fatty-acid-binding protein, alongside eGFR and serum creatinine measurements [35,46,47,48].

## 9. Future Perspectives

Whilst NGAL has shown promise as a potential biomarker, the majority of the available literature is based on single-centre prospective observational studies, and as such, there is a high risk of confounding. A 2019 meta-analysis by Li et al. has identified 14 observational studies in total and no randomised studies, of which only 4 scored for low risk of bias across all domains when evaluated with the QUADAS-2 tool [49]. Similar to the results reported by the studies in this review, their pooled findings supported diagnostic utility for both urinary NGAL (OR: 17.91 [95%CI: 7.22–44.43]; sensitivity: 85% [95%CI: 71–93%]; specificity: 80% [95%CI: 69–88%]) and plasma NGAL (OR: 43.11, [95%CI: 16.43–113.12]; sensitivity 91% [95%CI: 81–96%]; specificity 86%, [95%CI: 78–92%]) respectively; however, they identified significant between-study heterogeneity in their pooled analysis. It is likely that residual confounding, alongside variation in cut-off thresholds for NGAL, methods and time points for measuring NGAL, variation in definition of DGF, as well as patient and hospital-level factors, is driving between-study heterogeneity. In order to address this, future meta-analyses should use meta-regression techniques to explore heterogeneity more closely. Multicentre prospective studies are indicated to confirm the findings evaluated in the literature thus far and should consider auditing 1-year outcomes to establish whether there is a definitive correlation between NGAL and long-term graft survival and function. However, if such studies demonstrate causal associations, what is of crucial clinical importance is how clinicians use NGAL measurements to change management and improve outcomes. Future studies need to assess whether measuring NGAL will lead to timely intervention for delayed graft function and whether measurement within this timeframe ultimately impacts patient outcomes. Therefore, whether detection and prognostication of DGF based on NGAL will improve outcomes is still up for debate. If causal associations are supported, it would also be prudent for future studies to assess whether integration of NGAL measurement into a risk score can further enhance prediction of DGF.

## 10. Drawbacks of Using NGAL as a Marker

Utilisation of NGAL itself is not without limitations. As authors have previously reported, the NGAL assay ranges from £24 to £27 per test, which is more expensive than assaying serum creatinine alone (around £2 per test) [50]. Therefore, a cost–benefit analysis would be important in determining whether NGAL offers benefit beyond other biomarkers. Moreover, NGAL may be elevated by other causes; Cantaluppi et al. observed that mean NGAL levels were significantly different before and after tacrolimus administration (107.9 ± 37.66 ng/mL versus 155.9 ± 48.3 ng/mL, *p* < 0.001) [29]. Therefore, tacrolimus toxicity may falsely elevate NGAL levels. Moreover, in the presence of haematuria, which can be observed following transplantation or as a result of infection, detection of NGAL may be affected due to haemoglobin interacting with assays [28]. Therefore, interpretation of NGAL may be limited in certain clinical contexts. Finally, measuring urine NGAL may be challenging in the context of delayed graft function primarily because the graft itself is often anuric in the most severe instances of delayed graft function.

The data comparing biomarkers are limited to a small number of observational studies, which measure NGAL in different contexts; Sun et al. compared perfusate NGAL, KIM-1, IL-18, and L-FABP in 102 kidney transplant recipients [51]. Only NGAL and KIM-1 were significantly associated with DGF (both *p* < 0.001) and both remained independent predictors on multivariate analysis, with near-identical diagnostic performance (NGAL: AUC 0.83; KIM-1: AUC 0.82). IL-18 and L-FABP showed no predictive value. At a 3-year follow-up, both NGAL and KIM-1 correlated with reduced eGFR. This supports NGAL and KIM-1 as comparably strong DGF biomarkers, with IL-18 and L-FABP offering little added value in this cohort. Maslauskiene et al. compared pretransplant donor NGAL, KIM-1, IL-18, and CXCL10 in 43 donors/76 recipients [52]. Serum IL-18 and urinary KIM-1, not NGAL, were the independent multivariate predictors of DGF (AUROC 0.863); donor serum NGAL was not significant (*p* = 0.150). For long-term outcomes, urinary KIM-1 was the most consistent predictor of reduced eGFR through 3 years, while NGAL’s predictive value varied by compartment (serum vs. urine) and time point. This donor-biomarker study suggests KIM-1 and IL-18 outperformed NGAL for DGF prediction specifically, reinforcing that no single biomarker shows consistent superiority across contexts.

A further consideration is that long-term graft decline is unlikely to be driven by a single acute-injury event alone. Chronic kidney disease, including in the transplant setting, is increasingly understood as a state of persistent, low-grade inflammation, in which gut dysbiosis and intestinal barrier dysfunction promote metabolic endotoxemia and act as an upstream driver of systemic inflammatory signalling, partly via NLRP3 inflammasome-dependent maturation of IL-18 [53]. While this mechanism has not been directly tested in the kidney transplant NGAL literature, it raises the hypothesis that IL-18, unlike NGAL, may be mechanistically coupled to chronic, gut microbiome-driven inflammatory pathways in addition to acute tubular injury—a distinction that could plausibly contribute to the differing long-term performance of these biomarkers observed by Maslauskiene et al., though this remains speculative and warrants direct investigation. This limitation implies the use of a single biomarker that may not be coupled to the gut–kidney axis may not be sufficient in predicting long-term graft dysfunction.

## 11. NGAL’s Role in the Donor

Whilst this review has largely focused on the role of NGAL in the recipient, an emerging area of interest is whether donor NGAL can predict outcomes. A 2011 study assessed whether donor urinary NGAL could predict delayed graft function and reported an AUC of 0.595 (95%CI: 0.506–0.749) on multivariate regression [54]; urinary NGAL similarly showed an AUC of 0.616 (95%CI: 0.493–0.739) for prolonged DGF [54]. These AUC values are modest and, by conventional benchmarks, indicate poor discriminatory performance—only marginally better than chance—and should not be interpreted as evidence that donor NGAL offers clinically meaningful predictive utility in its current form. Furthermore, a 2018 study assessing the relationship between donor and recipient NGAL and recipient creatinine found no significant association (*p* > 0.3) [55]. Taken together, the current evidence for donor NGAL as a predictor of recipient outcomes is weak and inconsistent; while the concept merits further investigation, existing data do not support donor NGAL as a reliable or clinically actionable marker at this time, and conclusions in this area should be interpreted with caution.

## 12. Limitations of the Available Literature

This review is not without limitations; the majority of studies included in this review were single-centre and observational in nature. The small sample sizes and heterogeneity between patient populations limit the conclusions that can be drawn from this analysis of the available evidence. The nature of the single-arm cohort study design and the absence of comparison of NGAL with other predictors of graft outcomes, namely trends in eGFR, creatinine and other biomarkers such as KIM-1 and clinical determinants such as donor age, ischaemia time and recipient comorbidity burden, means that NGAL cannot be demonstrated with high certainty to be superior to other indicators of graft function, tempering the conclusions that can be drawn about this biomarker.

Moreover, differences in donor types (such as living and brain-dead donor), peri-operative care, and duration of the included studies, further compound heterogeneity between outcomes. Differences in donor type—living donor, donation after brain death (DBD), and donation after circulatory death (DCD)—are associated with distinct baseline ischemic injury profiles, and DCD grafts in particular are exposed to a period of warm ischemia that is known to independently elevate biomarkers of tubular injury, including NGAL, irrespective of eventual graft function. Similarly, variation in peri-operative care—namely cold and warm ischemia times, perfusion technique (static cold storage versus hypothermic or normothermic machine perfusion), and induction/maintenance immunosuppression protocols—may independently influence NGAL kinetics and confound cross-study comparison. Studies that fail to account for differences in donor type and perioperative care risk conflating the biomarker signal of ischemia–reperfusion injury with that of clinically meaningful graft dysfunction. Future publications could consider stratifying NGAL assessment by patient and donor characteristics as well as for variations in perioperative care, which may allow for a more granular assessment of where NGAL may best be poised.

DGF was not consistently defined across studies—whilst the conventional dialysis-within-7-days definition was most common, several studies used alternative or composite definitions incorporating creatinine reduction ratio or duration thresholds, which are known to yield materially different diagnostic accuracy estimates for the same biomarker. Compounding this, NGAL itself was measured using a range of assay platforms (including point-of-care immunoassays and laboratory-based ELISA), sample types (serum, plasma, and urine), and timing protocols, with no consensus cut-off value applied across studies. This lack of assay and threshold standardisation may explain the high pooled heterogeneity in pooled analyses [48].

Another limitation of this review is the vulnerability of using diagnostic performance estimates. Measurements of area under the curve, sensitivity, and specificity may indicate a correlation between NGAL levels and the appropriate outcome measure being studied. The rationale for measuring a biomarker that can predict poor graft function is compelling. However, these measurements do not necessarily indicate clinical benefit; to date, none of the included studies were designed to evaluate whether NGAL-guided decision-making—for example, earlier initiation of dialysis, modification of immunosuppression, closer monitoring, or altered surgical or perioperative management—translates into improved graft or patient outcomes. In the absence of such data, it remains uncertain whether identifying a patient as a high-risk patient through NGAL changes what a clinician would otherwise do or simply confirms a diagnosis of impaired graft function that would in most cases become clinically apparent shortly thereafter through rising serum creatinine or oliguria. Until an association with improved patient outcomes is demonstrated, NGAL measurement can only be utilised as a prognostic marker rather than an intervention that can improve patient care.

## 13. Conclusions

In summary, Neutrophil gelatinase-associated lipocalin is a promising investigational biomarker. Its potential benefits include timely, accurate detection of DGF, prediction of duration of DGF and prediction of long-term graft outcomes. Current limitations include the lack of high-evidence literature available outside of single-centre prospective observational studies, limitations secondary to cost and the lack of consensus on cut-off thresholds for measurement, alongside limited evidence demonstrating its adoption and utility in risk scores and combined assays. The lack of high-level evidence alongside the impact of its interpretation on clinical management currently limits the conclusions that can be drawn from its use in clinical practice. Further research should contextualise NGAL with predefined thresholds, multicentre validation and demonstrate its impact on clinical outcomes to fully unlock its potential benefit in transplant surgery.

## Data Availability

No new data were created or analyzed in this study. Data sharing is not applicable to this article.
